# Acoustic Analysis of Speech for Screening for Suicide Risk: Machine Learning Classifiers for Between- and Within-Person Evaluation of Suicidality

**DOI:** 10.2196/45456

**Published:** 2023-03-23

**Authors:** Sooyeon Min, Daun Shin, Sang Jin Rhee, C Hyung Keun Park, Jeong Hun Yang, Yoojin Song, Min Ji Kim, Kyungdo Kim, Won Ik Cho, Oh Chul Kwon, Yong Min Ahn, Hyunju Lee

**Affiliations:** 1 Department of Neuropsychiatry Seoul National University Hospital Seoul Republic of Korea; 2 Department of Psychiatry Seoul National University College of Medicine Seoul Republic of Korea; 3 Department of Psychiatry Korea University College of Medicine Seoul Republic of Korea; 4 Department of Psychiatry Asan Medical Center Seoul Republic of Korea; 5 Department of Biomedical Engineering Duke University Durham, NC United States; 6 Department of Electrical and Computer Engineering Seoul National University Seoul Republic of Korea; 7 MDB Incorporated Seoul Republic of Korea

**Keywords:** suicide, voice analysis, mood disorder, artificial intelligence, screening, prediction, digital health tool

## Abstract

**Background:**

Assessing a patient’s suicide risk is challenging for health professionals because it depends on voluntary disclosure by the patient and often has limited resources. The application of novel machine learning approaches to determine suicide risk has clinical utility.

**Objective:**

This study aimed to investigate cross-sectional and longitudinal approaches to assess suicidality based on acoustic voice features of psychiatric patients using artificial intelligence.

**Methods:**

We collected 348 voice recordings during clinical interviews of 104 patients diagnosed with mood disorders at baseline and 2, 4, 8, and 12 months after recruitment. Suicidality was assessed using the Beck Scale for Suicidal Ideation and suicidal behavior using the Columbia Suicide Severity Rating Scale. The acoustic features of the voice, including temporal, formal, and spectral features, were extracted from the recordings. A between-person classification model that examines the vocal characteristics of individuals cross sectionally to detect individuals at high risk for suicide and a within-person classification model that detects considerable worsening of suicidality based on changes in acoustic features within an individual were developed and compared. Internal validation was performed using 10-fold cross validation of audio data from baseline to 2-month and external validation was performed using data from 2 to 4 months.

**Results:**

A combined set of 12 acoustic features and 3 demographic variables (age, sex, and past suicide attempts) were included in the single-layer artificial neural network for the between-person classification model. Furthermore, 13 acoustic features were included in the extreme gradient boosting machine learning algorithm for the within-person model. The between-person classifier was able to detect high suicidality with 69% accuracy (sensitivity 74%, specificity 62%, area under the receiver operating characteristic curve 0.62), whereas the within-person model was able to predict worsening suicidality over 2 months with 79% accuracy (sensitivity 68%, specificity 84%, area under receiver operating characteristic curve 0.67). The second model showed 62% accuracy in predicting increased suicidality in external sets.

**Conclusions:**

Within-person analysis using changes in acoustic features within an individual is a promising approach to detect increased suicidality. Automated analysis of voice can be used to support the real-time assessment of suicide risk in primary care or telemedicine.

## Introduction

### Background

Suicidal behavior is a major public health problem that causes death in >700,000 people annually [1]. Suicidal ideation, defined by the National Institute of Mental Health as “thinking about, considering or planning suicide,” is one of the strongest predictors of suicidality. Therefore, understanding suicidal ideation is key to suicide prevention [2,3]. However, assessing suicidal ideation is challenging for health professionals because it depends largely on voluntary disclosure by the patient. According to a literature review, about half of patients with suicidal tendencies denied suicidal ideation during conversations in the week or month before their suicide [4]. Assessment becomes more difficult for nonspecialists, including emergency physicians, who have less experience interviewing psychiatric patients but are also under great time pressure [5,6]. Although numerous scales for suicidal risk assessment have been developed to overcome these limitations, both self-report such as the Beck Scale for Suicide Ideation and clinician-administered scales such as the Columbia-Suicide Severity Rating Scale (C-SSRS) have demonstrated limited predictive validity [7,8]. These conventional scales also require some time investment [9] and remain dependent on the participant’s willingness to disclose suicidal ideation.

Language and speech are the most important sources of data for the diagnosis of mental disorders [10]. In addition to speech content, vocal characteristics have historically been useful indicators of a patient’s mental state. In 1921, Emil Kraepelin described depressed voices as voices with a lower pitch, lower intensity, slower tempo, and monotone [11]. Although mental health assessments usually require face-to-face interviews, in some emergencies, they must be conducted using only voice. For example, in South Korea, the Korea Suicide Prevention Center has implemented 24-hour telephone counseling for people in distress or in need of emotional support as one of the strongest suicide prevention measures [12]. For counselors on such crisis hotlines, voice is the only source of information against which suicide risk must be assessed. With advances in artificial intelligence and its increasing application in medicine, quantitative analysis of the human voice using machine learning classifiers or deep learning architectures has become possible [13]. This is emerging as an innovative tool for screening for psychiatric symptoms. Studies have shown that various features of voice, such as pitch and speech rate, can be sensitive and valid measures of mood changes ranging from depression to mania [14,15]. Faurholt-Jepsen [16] reported that the acoustic features of voice can classify manic or mixed states with an area under the curve (AUC) of 0.89 and depressive states with an AUC of 0.78. In South Korea, voice analysis using a multilayer processing machine learning method could distinguish between mild and severe depression from euthymia with an AUC of 0.66 [17]. Notably, Costantini et al [18] demonstrated the potential of machine learning algorithms to develop universal emotion detection systems that are robust to variations in language, sex, and culture [18].

Many studies have also quantified the acoustic features of suicidal speech [19,20] and used machine learning methods for classification [21-25]. Recently, Belouali et al [23] classified suicidal utterances using the acoustic features of audio recordings of US veterans with 73% accuracy. However, these studies were only designed to examine suicide risk in a cross-sectional manner. Suicidal ideation is phenotypically heterogeneous and varies in duration, frequency, quality, and severity within and between individuals. The literature suggests that a dichotomous classification of suicide risk—low versus high suicide risk—at the population level is inadequate for predicting suicide risk, and longitudinal methodologies are needed to detect within-person changes [26]. There is evidence of different subtypes based on changes in suicidal ideation over time, underscoring the importance of understanding the time course of suicide risk in individual [27-29].

### Objective

In this prospective study, we collected voice recordings from 104 patients diagnosed with mood disorders over a 1-year period. Our objective was to develop the following two classification models of suicidality based on acoustic features of the voice: (1) a between-person (or interindividual) model that compares one’s own recording with other patient recordings and classifies whether the patient is at high risk for suicide and (2) a within-person (or intraindividual) model that compares an individual patient’s recordings and classifies whether their suicidality has significantly worsened since the last visit. We hypothesized that the within-person model would classify increased suicidality in participants with higher accuracy than the between-person model.

## Methods

### Study Participants and Setting

Adult patients (aged ≥19 years) who visited the Mood and Anxiety Clinic at Seoul National University Hospital between January 2019 and July 2021 were recruited using convenience sampling. Diagnoses were made using Mini-International Neuropsychiatric Interview version 7.0.2. Patients with bipolar disorder (all types, including I, II and Not Otherwise Specified) or major depressive disorder, according to the Diagnostic and Statistical Manual of Mental Disorders, Fifth Edition [30], were included in the study. Patients diagnosed with other major psychiatric disorders such as psychotic and neurocognitive disorders were excluded. Participants were also excluded if their voices were affected by laryngeal disease or surgery. Participants whose psychiatric symptoms could be affected by organic brain diseases, such as epilepsy, traumatic brain injury, or multiple sclerosis, or who had undergone intracranial surgery were excluded from the study.

We initially aimed to recruit 100 patients with mood disorders based on previous voice analysis studies that used machine learning to analyze voice characteristics to detect mood changes or suicidality [23,31,32]. We also aimed to recruit 100 healthy controls through posts and web-based advertisements. Healthy controls were excluded if they had been diagnosed with any psychiatric disorder, laryngeal condition that affected their voice, or other neurological conditions that could cause psychiatric symptoms. However, after conducting an exploratory analysis at baseline, healthy controls were excluded from the main analyses to ensure homogeneity of the study population and to avoid potential confounding. Further details regarding this decision are provided in the *Discussion* section.

At their first visit, study participants completed a written informed consent form. The patients underwent clinical examination and completed self-reported questionnaires at recruitment and follow-up visits (at 2, 4, 8 and 12 months). Voice was recorded during each visit in a private room. Only the numerical data of the clinical variables and acoustic features of the study participants were used in the analysis, making it impossible to identify the speaker. The raw files of voice recordings were anonymized, kept locked, and were not to be used for any purpose other than the study consented by the study participants. All procedures followed the rules introduced by the amendment to the Personal Information Protection Act [33].

### Clinical Assessments

Demographic data, such as sex, age, socioeconomic status (SES), medication use, height, weight, and concomitant medical conditions were collected. As the voice can be affected by antipsychotics [34], the total dose of antipsychotics was summed after being converted to the equivalent dose of aripiprazole [35], which was the most commonly prescribed drug in our study population. At each visit, independent raters assessed the severity of suicidal intent and other psychiatric symptoms using the Korean versions of the C-SSRS and the Hamilton Depression Rating Scale (HDRS). Training sessions and regular monitoring of ratings were conducted to ensure an objective and unbiased rating. Patients also self-rated their suicidality, symptoms of depression and anxiety, and impulsivity using the Korean version of Beck’s Scale for Suicidal Ideation (SSI), Patient Health Questionnaire-9 (PHQ-9), Beck Anxiety Inventory (BAI), and Barratt Impulsiveness Scale (BIS).

To assess the severity of suicidality, we selected the C-SSRS, considered the gold standard for measuring suicidal ideation and behavior by the US Food and Drug Administration [36], and the SSI, which is the most commonly used self-reporting scale for assessing suicidal ideation and has been shown to be valid for longitudinal measurement [37]. The C-SSRS is a semistructured interview that assesses the presence and severity of suicidal ideation and behavior. The 2 constructs measured the severity and intensity of suicidal ideation on a 5-point ordinal scale. The third construct assessed the number of actual, interrupted, and aborted suicide attempts and the presence of nonsuicidal self-injurious behaviors. The final construct is the actual attempt lethality subscale, which is answered only when there has been an actual suicide attempt [38]. On this subscale, the interviewer records the date of the most lethal attempt and rates the actual lethality or medical damage, which ranges from 0 (no physical damage or very minor physical damage, such as surface scratches) to 5 (death). If the actual lethality is 0, the interviewer rates the potential lethality of the suicidal behavior, ranging from 0 (behavior not likely to result in injury) to 2 (behavior likely to result in death despite available medical care). The SSI, described below, is a 19-item self-report inventory that quantifies suicidal intent [39]. To evaluate the severity of depressive symptoms related to suicidality and changes in voice, the HDRS and PHQ-9, the most commonly used symptom severity scales to assess depressive symptoms in longitudinal studies [40,41], were used. The HDRS is a 17-item clinician-administered scale used to assess depressive symptoms. Clinicians rated each item on a scale from 0 (not present) to 4 (severe). Scores higher than 16 indicated moderate depression and scores higher than 24 indicated severe depression [42-44]. The PHQ-9 is a self-administered scale used to assess subjective depression. Each of the 9 items is rated on a 5-point ordinal scale, with scores of ≥10 points indicating moderate-to-severe depression [45]. The BAI and BIS questionnaires were used to assess the levels of anxiety and impulsiveness, respectively, which are associated with suicidality. The BAI is a self-administered scale to assess subjective anxiety and consists of 21 items scored on a 4-point ordinal scale. A score of ≥19 indicates moderate anxiety [46]. The BIS is a self-administered scale measuring impulsiveness and consists of 30 items scored on a 5-point ordinal scale [47].

### Voice Features

During each visit, the participants’ voices were recorded while talking with 1 of the 2 trained research nurses using a Sony ICD-SX813 voice recorder and saved in MP3 format, with a bit rate of 128 kbps, a bit depth of 32 bits, and a sampling rate of 44.1 kHz. The recordings were conducted in a single interview room, with the participant speaking from a distance of 60 to 100 cm from the recorder. Although there may have been unexpected background noise, such as hospital announcements, during the preprocessing stage, we only extracted voice segments that did not contain mixed noise. Utterances shorter than 3 seconds were excluded, and the remaining utterances were segmented into 10-second lengths.

Temporal, formant, spectral, and other physical features of the voice were extracted for each segmented utterance and averaged over the entire time interval [17]. Temporal features include the timing and length of the utterance interval and tempo (ie, the degree of periodicity of the acoustic components). The spectral features included the averaged spectral centroid, spectral bandwidth, roll-off frequency, root-mean-square energy, and Mel-Frequency Cepstral Coefficients. The formant features obtained using the linear prediction coefficients are the local maxima of the spectrum and represent the resonance of the vocal tract. The first 3 formants and their corresponding bandwidths were extracted. Other physical attributes, including the mean and variance of the pitch, magnitude, zero-crossing rate, and voice components, were also extracted. In total, 60 acoustic features were extracted. Extraction was performed using Python (version 3.7) and the following packages were used to extract the acoustic features: librosa 0.7.2, NumPy 1.17.2, and pandas 1.4.1. Normalization or filtering was not performed during the preprocessing stage.

### Statistical Analysis

Patients were classified as being at high risk of suicide based on their SSI scores. The SSI was used for the following reasons: (1) earlier studies have established its high internal consistency, with a high Cronbach α ranging from 0.8 to 0.9, test-retest stability, and concurrent validity [39,48,49]; (2) SSI is modeled on the interviewer-rated Scale for Suicide Ideation, which is one of the few suicide scales with reported predictive validity for completed suicide [50]; and (3) SSI has been proven to be measurement-invariant over time, which is of particular importance for our longitudinal analysis [37]. In fact, a systematic review of the most commonly used suicide assessment instruments concluded that the SSI is one of the most reliable instruments for its psychometric properties, clinical utility, and cultural appropriateness [51]. The scale does not have a universal cutoff value for defining the severity of suicide risk, but previous studies in the Korean population have suggested the following clinical cutoffs: normal, 0-8; mild, 9-11; moderate, 12-14; and severe, 15-38 [52,53]. For between-individual analysis, patients with SSI ≥15 were classified as being severely suicidal. To our knowledge, there is no consensus regarding the definition of clinically important worsening or relapse of suicide risk in patients. For other psychiatric symptoms, such as psychosis, a variety of different criteria have been used to define relapse in previous studies [54,55], including quantitative criterion such as an increase in clinical scale score (eg, ≥25% increase in the Positive and Negative Syndrome Scale), and qualitative criteria such as psychiatric hospitalization and suicidal or self-injurious behavior [54]. On the basis of a previous longitudinal analysis using SSI, which concluded that changes in SSI scores over time could be attributed to true changes in suicidality [37], we defined “clinically significant worsening of suicide risk” as a ≥25% increase in SSI score or commitment to suicide attempt or self-injurious behaviors during the follow-up interval using the third construct of the C-SSRS.

The Student *t* test and chi-square test for independence were used to assess any differences in the demographic and clinical characteristics between patients at high risk for suicide and low risk of suicide. The Fisher exact test was used for comparisons with a small sample size. The level of significance was set at *P*<.05. For 25 voice recordings with incomplete SSI, SSI scores were imputed using multiple imputations (R package, *mice*), which is known to show the best performance in imputing missing values in self-questionnaires measuring psychiatric symptoms [56]. Multiple imputation uses covariates to generate a range of plausible values for each missing value through iterations [57]. Acoustic characteristics between the suicidal and nonsuicidal groups were compared using the 2-tailed *t* tests or Mann-Whitney *U* tests. All statistical analyses were performed using R (version 4.1.0; R Foundation for Statistical Computing).

To build a prediction model using machine learning, several classification algorithms have been investigated, including those already used in voice analysis, namely, the support vector machine (SVM), random forest (RF), light gradient boost (light GBM), extreme gradient boost (XGB) algorithms, and artificial neural networks (ANN) [16,23,53]. SVM uses hyperplanes to represent data points and attempts to maximize the margin between different classes. RF is defined as a multiple random decision tree structure based on bagging. Both methods are widely used in multiple machine learning tasks, including clinical data, owing to their simplicity. Light GBM and XGB are advanced machine learning algorithms. Light GBM is based on gradient boosting and has higher efficiency. XGB is also based on a boosting algorithm and is renowned for its fast convergence speed. An ANN uses single or multiple layers, including a fully connected layer. Various features are used as the data passes through these layers, we can use various features.

The prediction performance was evaluated based on the classification accuracy, which is the ratio of the number of correct predictions to the total number of input samples. The features used in the model were selected based on their clinical importance and statistical association with suicide risk or exacerbation through feature ranking [58]. For internal validation, *K*-fold cross-validation was performed. In this approach, “k” subsamples were randomly created from a data set and used separately to train (*k*-1) and test (1) the algorithm. This method is popular in machine learning studies using clinical data. It minimizes overfitting while overcoming the small sample size during repeated internal validations throughout the iterations [59]. Hyperparameter tuning was performed using Bayesian optimization [56], and the Synthetic Minority Oversampling Technique was used to account for class imbalance. Machine learning analyses were performed using Python 3.7.0, *scikit-learn* package 0.22, and *xgboost* package 0.90.

**Figure 1:**
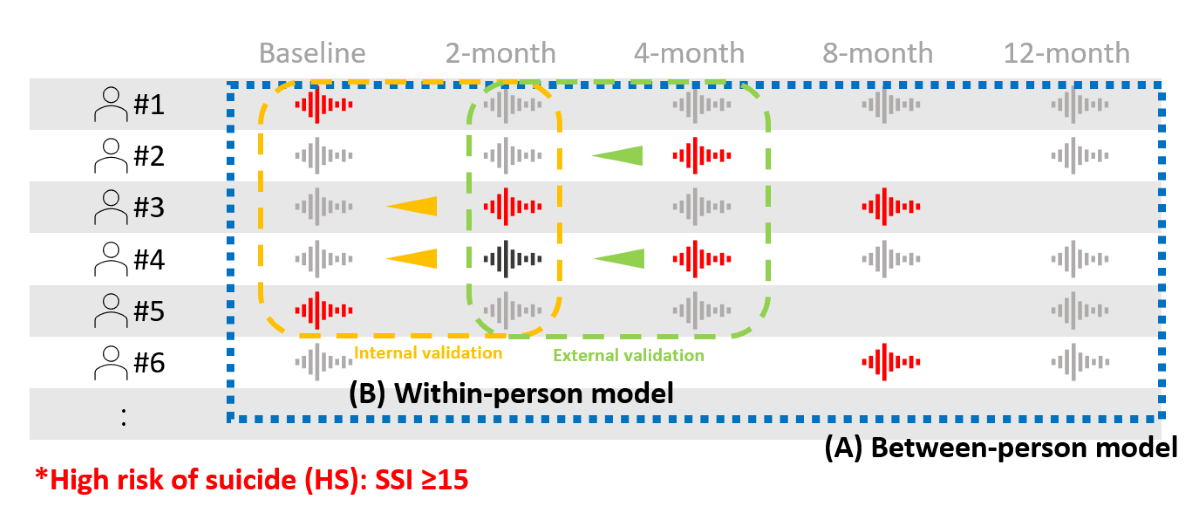
Schematic diagram representing the between-person and within-person classification models for suicide risk. (A) For the between-person model, voice recordings from high-risk suicide patients (color-coded red) were discriminated among all voice recordings. (B) The within-person classification model aimed to detect a clinically significant worsening of suicidal risk in a patient (represented as arrowhead) using the changes in the subjects’ auditory characteristics. The internal validation consisted of 10-fold cross-validation using data from patients at baseline and 2-month, and the external validation consisted of testing the same model using data from patients at 2 and 4 months.

Initially, the between-person classification model focused on discriminating between high- and low-risk suicide patients based on the acoustic features of their voices. Voice recordings of all the patients were performed using the same model. Next, the within-person classification model was concerned with the ability to detect clinically major worsening of suicidal ideation in patients. In this case, the changes in the participants’ auditory characteristics were analyzed. The data from baseline to 4-month were used in this analysis (Figure 1). To assess the performance of the models, we used *k*-fold cross-validation (*k*=10) within the data from participants at baseline and 2 months after recruitment as internal validation. For external validation, we evaluated the performance of the same model using the data from the 2- and 4-month assessments.

### Ethics Approval

All procedures were approved by the institutional review board of Seoul National University Hospital (1812-081-995) and performed in accordance with the ethical standards laid down in the 1964 Declaration of Helsinki and its later amendments.

## Results

### Demographic and Clinical Characteristics of Patients

A total of 104 participants diagnosed with mood disorders were included in the study. Of the 104, 12 (11.5%) patients were diagnosed with major depressive disorder and 92 (88.5%) with bipolar disorder. Furthermore, 76% (79/104) patients were female. Furthermore, 14.4% (15/104) of participants had a history of attempting suicide. At baseline, excluding the 6 study participants whose SSI was incomplete, 69% (68/98) participants were classified as having a high suicide risk (HS) based on their SSI. The patients in the HS group were younger (*P*=.01) and more likely to live alone (*P*=.049). They also suffered from more severe depressive symptoms based on the PHQ-9 (*P*<.001), HDRS (*P*<.001), and anxiety (*P*<.001) and were more likely to have a history of suicide attempts (*P*=.02). There were no statistically significant differences in sex, BMI, psychiatric diagnosis, SES, years of education, antipsychotic dosage, medical comorbidities, and impulsiveness (Table 1).

A total of 348 recordings were made during the study period. During the follow-up, 74% (77/104) participants visited at 2 months, 61.5% (64/104) at 4 months, 51% at 8 months (53/104), and 48.1% (50/104) at 12 months after recruitment. A total of 25 of them were present during the interview where their audio recordings were collected but did not complete the SSI questionnaire. For these participants, multiple imputations were performed based on demographic and clinical characteristics, including other psychiatric scales such as the HDRS.

For within-person analysis, recordings from 77 participants were used for internal validation. During the initial follow-up, 7% (6/77) of patients had committed nonsuicidal self-injury, and 9% (7/77) patients attempted suicide. Including patients who showed self-injurious or suicidal behaviors, a total of 28.6% (22/77) of patients were classified as having a clinically worsened risk for suicide. For external validation, 55 patients were followed up at both 2 and 4 months after recruitment, of whom 30.9% (17/55) were classified as having a clinically worsened suicide risk. The mean prescribed dose of antipsychotics at recruitment was equivalent to 12.35 mg of aripiprazole, whereas at 2 and 4 months after recruitment it was equivalent to 10.99 mg and 13.59 mg of aripiprazole, respectively. There was a statistically significant difference in the change in the prescribed dose of antipsychotics during the first 2 months between the patients whose suicide risk had worsened and the rest of the patients. The patients whose suicidal ideation did not worsen during the first 2 months were prescribed a lower dose of antipsychotics at the 2-month time point, the mean change was equivalent to −3.84 (SD 11.80) mg of aripiprazole, whereas the patients whose suicidal risk had worsened were prescribed a higher dose –that is, a 2.71 (SD 12.35) mg higher dose of aripiprazole (*t*_37_=2.13; *P*=.04). However, there was no significant difference in the change in the prescribed dose of antipsychotics between the 2 groups during the next 2 months, that is, from 2 to 4 months after recruitment (*t*_48_=−0.23; *P*=.82).

**Table 1:** Baseline characteristics of the high suicide and low suicide risk groups.

Characteristics		High risk of suicide	Low risk of suicide	*P* value	
Number of participants, n (%)		68 (69)	30 (31)	N/A^a^	
Age in years, mean (SD)		28.53 (9.08)	34.70 (11.67)	.01	
**Sex, n (%)**					.97
	Male	14 (21)	7 (23)		
	Female	54 (79)	23 (77)		
BMI, mean (SD)		24.30 (4.97)	25.09 (5.01)	.47	
**Diagnosis, n (%)**					.99
	Bipolar disorder	60 (88)	26 (87)		
	Major depressive disorder	8 (12)	4 (13)		
**Marital status, n (%)**					.049
	Single	54 (79)	18 (60)		
	Married	10 (15)	11 (37)		
	Divorced or widowed	4 (6)	1 (3)		
Household income^b^, median (range)		450 (50-5000)	530 (74-1000)	.79	
**SES^c^, n (%)**					.62
	Very low	1 (2)	2 (7)		
	Low	13 (19)	6 (20)		
	Middle	37 (54)	13 (43)		
	High	9 (13)	4 (13)		
	Very high	8 (12)	5 (17)		
Years of education, mean (SD)		13.94 (1.95)	14.53 (2.32)	.23	
Antipsychotics dosage^d^, median (range)		11.94 (0-85)	11.61 (0-70)	.92	
**Medical comorbidity, n (%)**					.35
	None	52 (77)	25 (83)		
	Mild	12 (18)	2 (7)		
	Moderate	3 (5)	3 (10)		
	Severe	1 (1.5)	0 (0)		
History of suicide attempt, n (%)		12 (17)	0 (0)	.02	
PHQ-9^e^, mean (SD)		17.66 (5.74)	10.67 (6.19)	<.001	
HDRS^f^, mean (SD)		18.37 (4.30)	13.83 (4.49)	<.001	
BAI^g^, mean (SD)		27.32 (16.57)	15.23 (13.62)	<.001	
BIS^h^, mean (SD)		63.40 (7.69)	62.70 (8.05)	.69	
SSI^i^, mean (SD)		23.60 (5.66)	7.07 (5.04)	<.001	

^a^N/A: not applicable.

^b^Unit=10,000 won.

^c^Socioeconomic class based on the Hollingshead and Redlich index.

^d^Antipsychotics dosage converted into dose equivalent of aripiprazole.

^e^PHQ-9: Patient Health Questionnaire-9.

^f^HDRS: Hamilton Depression Rating Scale.

^g^BAI: Beck Anxiety Inventory.

^h^BIS: Barratt Impulsiveness Scale.

^i^SSI: Beck Scale for Suicidal Ideation.

### Acoustic Analysis

The correlations between the characteristics of the 60 extracted acoustic features of voice and suicidality are presented in Table 2. Suicidal recordings differed from nonsuicidal recordings in root-mean-square energy, F1, magnitude error, MFCC1, MFCC5, MFCC22, and MFCC27. Suicidal recordings also showed a tendency toward a higher first-formant bandwidth and a lower mean pitch. For the within-person model, the change in acoustic features, that is, ∆_0→t_(acoustic feature), was measured from baseline to 2-month and from 2 to 4 months. Patients whose suicidality had worsened compared with 2 months earlier showed statistically significant changes in Mel-frequency cepstral coefficients (MFCC) MFCC8, MFCC29, MFCC30, MFCC31, and MFCC32. The Mel-frequency cepstral coefficients are coefficients derived by computing a spectrum of the log-magnitude Mel-spectrum of the audio segment; the lower coefficients represent the vocal tract filter, and the higher coefficients represent the periodic vocal fold sources [14].

**Table 2:** Acoustic features in the recordings from patients at high risk versus low risk of suicide; the between-person analysis.

	Suicidal recordings (n=192), mean (SD)	Nonsuicidal recordings (n=156), mean (SD)	*P* value (*t* test)
Time (seconds)	13.71 (7.27)	13.91 (7.52)	.82
spectral_centroid	7.37 (0.12)	7.37 (0.11)	.61
spectral_bandwidth	7.41 (0.08)	7.40 (0.08)	.54
spectral_roll-off	7.98 (0.17)	7.99 (0.16)	.81
Root-mean-square energy	4.26 (0.72)	4.06 (0.74)	.02
Tempo	118.52 (1.54)	118.65 (1.91)	.54
F0	6.23 (0.07)	6.23 (0.08)	.94
F1	7.31 (0.12)	7.34 (0.13)	.03
F2	8.01 (0.10)	8.01 (0.08)	.96
formant_bandwidth0	44.45 (14.22)	41.31 (14.15)	.05
formant_bandwidth1	205.43 (48.79)	202.78 (47.10)	.63
formant_bandwidth2	224.65 (42.92)	216.20 (42.10)	.09
mean_pitch	274.76 (31.98)	281.39 (27.73)	.05
error_pitch	0.33 (0.08)	0.32 (0.08)	.17
change_p	35.70 (19.12)	36.02 (23.85)	.90
mean_magnitude	61.36 (11.22)	62.66 (9.05)	.26
error_magnitude	0.85 (0.17)	0.89 (0.15)	.03
change_magnitude	66.77 (44.89)	67.69 (36.26)	.84
Zero-crossing rate	0.05 (0.01)	0.05 (0.01)	.23
Voice portion	0.68 (0.04)	0.69 (0.03)	.81
MFCC^a^1	−350.70 (66.61)	−331.03 (65.26)	.01
MFCC2	115.87 (11.73)	113.65 (10.52)	.08
MFCC3	−37.54 (8.90)	−37.58 (9.53)	.97
MFCC4	6.66 (6.10)	6.30 (6.28)	.62
MFCC5	−41.50 (6.18)	−39.77 (6.38)	.02
MFCC6	−15.07 (6.54)	−15.95 (7.59)	.29
MFCC7	−35.15 (4.70)	−35.64 (4.67)	.37
MFCC8	−24.57 (5.32)	−24.36 (5.34)	.74
MFCC9	−24.05 (4.04)	−24.01 (3.89)	.98
MFCC10	−20.08 (3.46)	−20.45 (3.50)	.37
MFCC11	−17.61 (3.32)	−17.44 (3.43)	.66
MFCC12	−15.37 (3.03)	−15.86 (3.20)	.18
MFCC13	−15.02 (3.00)	−14.56 (2.86)	.18
MFCC14	−11.90 (2.62)	−11.86 (2.68)	.92
MFCC15	−10.64 (2.78)	−10.03 (3.22)	.08
MFCC16	−10.89 (2.89)	−10.71 (2.77)	.58
MFCC17	−8.01 (2.25)	−7.76 (2.07)	.31
MFCC18	−6.30 (2.21)	−2.92 (2.56)	.17
MFCC19	−7.02 (2.34)	−6.91 (2.26)	.69
MFCC20	−5.95 (2.22)	−5.69 (2.11)	.31
MFCC21	−5.32 (1.77)	−5.05 (2.23)	.25
MFCC22	−4.39 (1.86)	−3.91 (1.49)	.01
MFCC23	−3.58 (1.57)	−3.76 (1.58)	.31
MFCC24	−2.11 (1.68)	−1.74 (1.85)	.08
MFCC25	−2.83 (1.49)	−2.69 (1.52)	.41
MFCC26	−1.49 (1.79)	−1.38 (1.76)	.59
MFCC27	−0.54 (1.83)	−0.08 (1.83)	.03
MFCC28	0.09 (1.80)	0.04 (1.73)	.80
MFCC29	0.42 (1.98)	0.50 (1.89)	.74
MFCC30	1.16 (1.96)	1.34 (2.05)	.45
MFCC31	1.82 (1.82)	2.08 (2.08)	.45
MFCC32	2.98 (1.95)	2.89 (2.05)	.70
MFCC33	3.31 (2.03)	3.25 (2.11)	.78
MFCC34	3.50 (1.98)	3.20 (1.77)	.17
MFCC35	2.96 (1.92)	2.71 (1.86)	.25
MFCC36	3.25 (1.85)	2.91 (1.74)	.11
MFCC37	3.07 (1.67)	2.93 (1.70)	.46
MFCC38	3.40 (1.74)	3.26 (1.57)	.45
MFCC39	2.63 (1.49)	2.58 (1.40)	.78
MFCC40	2.05 (1.42)	2.00 (1.42)	.78

^a^MFCC: Mel-frequency cepstral coefficient.

### Machine Learning Predictive Models

#### Between-Person Classification Model

**Figure 2:**
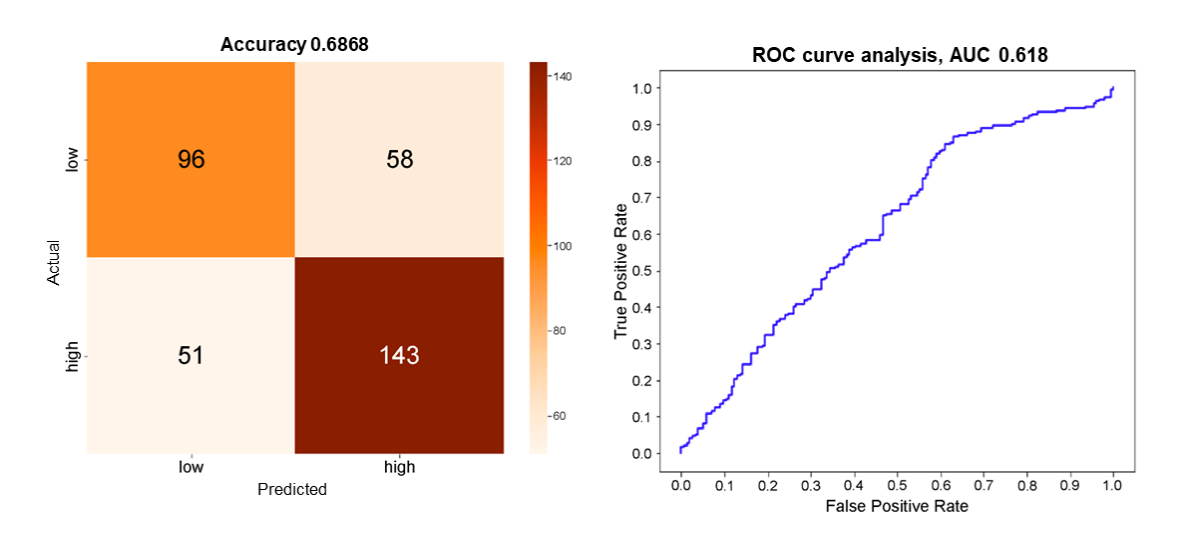
Confusion matrix and receiver operating characteristic (ROC) curve for between-person classification model based on a single layer artificial neural network. AUC: area under the ROC curve.

The demographic features included in the model were sex, age, and past suicide attempts. The voice features included in the model were the root-mean-square energy, F1, F0 bandwidth, F2 bandwidth, mean pitch, magnitude error, MFCC1, MFCC5, MFCC15, MFCC22, MFCC24, and MFCC27. The XGB classifier achieved 63.7% accuracy, whereas the light GBM had 61.1% accuracy. The RF and SVM classifiers did not perform better. The classification model based on an ANN with a single layer performed better, with an accuracy of 68.7% (Figure 2). The sensitivity (or recall) of the classification model was 73.7%, specificity was 62.3%, precision was 71.1%, and the *F*_1_-score was 72.4%. The AUC was 0.62.

#### Within-Person Classification Model

**Figure 3:**
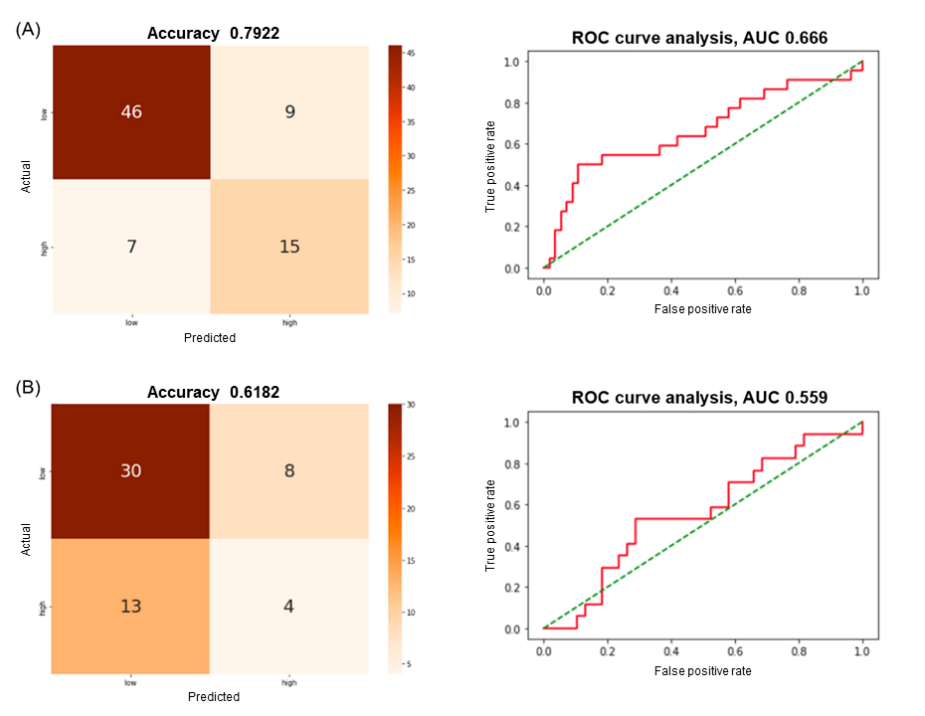
Confusion matrices and receiver operating characteristic (ROC) curves for within-person classification model based on extreme gradient boot classifier. (A) Performance of the within-person classification model to predict worsening of suicide risk from baseline to 2 months. (B) Performance of the same model in the external sets, that is, from 2 to 4 month. AUC: area under the ROC curve.

Voice features such as root-mean-square energy, MFCC1, MFCC8, MFCC9, MFCC19, MFCC20, MFCC28, MFCC29, MFCC31, MFCC33, MFCC34, MFCC35, and MFCC36, and the same demographic features as the between-person classification model were used. From baseline to 2 months, that is, internal validation, XGB showed the best performance, with an accuracy of 79.2%. The sensitivity of the model was 68.2%, its specificity 83.6%, precision 62.5%, and *F*_1_-score 65.2%. When the same model was applied for prediction from 2 to 4 months for external validation, the accuracy was 61.8%. The sensitivity of the model was 23.5%, specificity was 78.9%, precision was 33.3%, and *F*_1_-score was 56.8%. The AUC for internal validation was 0.67 and that for external validation was 0.56 (Figure 3).

## Discussion

### Principal Findings

In this prospective analysis of patients diagnosed with mood disorders using machine learning, within-person changes in voice features predicted worsening suicidality over 2 months with 79% accuracy. A cross-sectional analysis of 348 patient recordings achieved a 69% accuracy in detecting high suicide risk. Younger age, single status, past suicide attempts, and higher severity of depression and anxiety symptoms are associated with higher suicidality in patients with mood disorders. Single status, previous suicide attempts, and higher depression severity are known risk factors for suicide [50].

Suicidal recordings were associated with greater root-mean-square energy and SE of magnitude, and lower first formant frequency (F1) than those of nonsuicidal recordings. They also distinguished between MFCC1, MFCC5, MFCC22, and MFCC27. The recordings from participants at increased suicide risk showed changes in MFCC8, MFCC29, MFCC30, MFCC31, and MFCC32 compared with those 2 months earlier. These results are consistent with those of previous studies that concluded that formant behavior and power distribution appear to distinguish the acoustic characteristics of patients with major depression and high suicide risk [15,20]. Frances et al [20] reported that the root-mean-square and coefficient of variation of amplitude—comparable with the root mean square energy and SE of magnitude in our study—were among the optimal discriminators of suicidal and depressed speech. Although several studies have reported differences in the first formant frequency of depressive and suicidal speech, the direction of change has been inconsistent in previous studies [20,60,61]. This inconsistency can be partially explained by the different methods used to collect voice samples. Some studies were conducted in a laboratory setting, where a specific task was given and portions of speech were analyzed (eg, vowels in Tolkmitt et al [61]), whereas others were conducted in a more naturalistic setting, such as therapy sessions or telephone conversations between patients and psychiatrists, where the voice features were averaged over the time of speech during the recording [20,24]. In this study, voice recordings were made during the clinical interview.

The SSI, which is used to label suicidality, does not have a universal cutoff value for defining the severity of suicidality. In a prospective 20-year study of 6891 psychiatric outpatients, those who scored ≥3 on the SSI were approximately 7 times more likely to commit suicide than those who scored <3 at baseline [50]. A retrospective study of data from 366 patients treated by a psychiatric liaison service in a general hospital after a suicide attempt suggested a cutoff value of SSI ≥6 for the best classification accuracy [62]. Compared with these studies, the average SSI score in our study was much higher (mean baseline SSI=18.5). This difference may be attributed to the higher severity of the symptoms of the study participants, who were patients with mood disorders who visited the outpatient clinic of a tertiary hospital; or to the cross-national difference, as previous studies in the Korean population also reported higher SSI scores and suggested a cutoff value of SSI ≥15 to define severe suicide risk [52,53].

### Strengths

To the best of our knowledge, this is the first study to analyze changes in voice characteristics over time within an individual to predict whether suicidality has worsened during follow-up. Previous studies focused on the cross-sectional assessment of suicide risk at the population level and used a method comparable with the first model in our study, that is, the between-person classification model [14,20,21,23,24]. Although much of the variability in acoustic features between individuals is due to the characteristics of organs involved in the production of sounds, such as the vocal or nasal tract, lungs, and chest wall, within-person variability better reflects changes in an individual’s condition. Indeed, individually trained machine learning models have been developed and have shown good results in other areas of psychiatry and neurology, such as affective [16,63] and movement disorders [64]. Suicidality is a complex symptom that may present and develop differently from person to person [26,29], and a personalized approach seems appropriate. In our study, the within-person classification model outperformed the between-person classification model, which was consistent with our hypothesis.

Another strength of our study is the relatively large sample size and the use of multiple imputations for missing data. In longitudinal studies with multiple measurement time points, attrition is inevitable. Ignoring missing data introduces biases of unknown magnitude and direction [65]. Patients may refuse to continue participating in the study at any point during the follow-up, and their refusal may be associated with an improvement or worsening of the outcome of interest. Multiple imputation is a robust method for dealing with missing data in longitudinal studies with repeated measures of self-reported outcomes [66], especially when the ratio of variables to cases with complete data is less than 1:3 [67]. In this study, of the 348 visits in which audio recordings were made, the SSI self-questionnaires were not completed in 7.2% (25/348) of studies. For these visits, multiple imputations were performed based on demographic (age, sex, antipsychotic dosage, SES, etc) and clinical covariates, including clinician-administered scales, such as the HDRS and C-SSRS.

The use of patient voices recorded during the clinical interviews has several advantages. Voice recordings are closer to real life, allowing for automated quantitative analysis of voices in the future. The interview also allowed the voice to be recorded for a sufficient period (>15 minutes), unlike most previous studies that used audio recordings ranging from a few seconds to several minutes in length [23-25], and permitted the inclusion of colloquial and paralinguistic expressions that are informative for speech analysis [15,17]. In a recent study using the Emofilm database, which is a collection of short clips of movies in several languages, Costantini et al [18] explored and concluded that a cross-language classifier maintains a Thigh performance despite variations in languages and cultures. In our study, technical specifications were controlled using the same microphone model in identical outpatient clinic settings, reducing device- and context-dependent noise and reverberation changes [14]. Stasak and Epps [68] reported that variability in audio acquisition techniques between different devices can lead to undesirable variations in the performance of audio-based classification algorithms.

### Limitations

Our study has several limitations. First, for between-person analysis, recordings from different time points were analyzed as independent observations, although some recordings were repeated samples from the same patients collected at different time points (Figure 1). Because of the challenges associated with recruiting patients with suicidality and obtaining voice recordings from them, such analyses—longitudinal audio collection and comprehensive analysis of recordings as independent measures—are common. In one of the most recent studies using voice characteristics to screen for suicidal ideation, which had the largest sample size, Belouali et al [23] developed a classification algorithm using 588 audio recordings obtained from 124 US veterans. Faurholt-Jepsen et al [16] and Arevian et al [63] used hundreds of phone calls collected during a follow-up period from 28 and 47 patients, respectively, and treated the data from each phone call as an independent measure to develop a “user-independent” or “population-level trained model.”

Second, the use of the criterion of an increase in the SSI score of ≥25% within 2 months for within-person analysis is subject to caveats. Patients who initially achieved a low SSI score were more likely to be classified as having increased suicide risk than those who initially achieved a higher score. A hypothetical patient who achieved a score of 3 at baseline and a score of 4 at 2-month was classified as having worsened suicidality, but not a patient who achieved a score of 15 at baseline and a score of 18 at follow-up. This makes the classifier more sensitive in detecting increased suicide risk in patients with low initial suicidality. Of the 77 subjects included in the within-person classification model in our study, 54 (70.1%) were classified as having a baseline SSI score of ≥15 (ie, high risk) and 13 (24.1%) were classified as having worsened suicidality during the 2-month follow-up period, whereas 9 (39.1%) of the 23 patients (29.9%) whose baseline SSI score was <15 (ie, low risk) were classified as having worsened suicidality. To test for bias using the percentage increase criterion, a classification model was tested that defined worsening as an increase in score of ≥1 on the SSI compared with 2 months earlier. Although 41.6% (32/77) of patients showed an increase in the SSI score of ≥1 at 2 months compared with baseline, the best classifier accuracy was only 67.5%, despite the mitigated class imbalance.

Third, potential confounders such as demographic factors (sex, age, etc) and antipsychotic medication use could not be controlled. Therefore, it is difficult to determine whether the changes in acoustic characteristics were solely because of changes in suicidal ideation, or were related to changes in antipsychotic dosage or other confounders. In particular, females outnumbered males in our study population, and the suicidal group was younger compared with the nonsuicidal group (Table 1). By limiting the study population to patients diagnosed with mood disorders and, thus, homogenizing the population, we were able to reduce some of the potential confounding factors. As a preliminary analysis, we developed an RF classifier algorithm using 100 patients diagnosed with mood disorder and 88 healthy controls, which correctly identified the patients with high suicide risk with an accuracy of 71% and an AUC of 0.69 (Multimedia Appendix 1). Although the classifier performed well, when we included healthy controls, we found that the suicidal group had a higher BMI and was prescribed a higher dose of antipsychotics than the nonsuicidal group (Multimedia Appendix 2). These differences may have contributed to the differences in acoustic features and acted as confounders in the analysis. Previous studies have suggested that BMI [69,70] and antipsychotic prescription [34] can cause changes in vocal cord tension and airflow, which in turn can affect measured acoustic features.

Fourth, despite the high accuracy, the AUC of the between-person model and that of the within-person model were only 0.62 and 0.67, respectively, which can be partially explained by the class imbalance and implies the importance of selecting an appropriate decision threshold [71]. Low AUC values suggest that the results may not be generalizable to larger populations. Fifth, the predictive ability of the classification model depends on the limited predictive validity of the SSI used for labeling. Although no assessment scale can perfectly predict suicidal behavior, the SSI is modeled on the interviewer-rated scale for suicide ideation, which is notably among the few suicide scales with reported predictive validity [37,50]. In addition, the SSI demonstrated strong correlations with other scales assessed in our study, including the C-SSRS and the item on the HDRS that assesses suicide risk (Item 3). The results of the Pearson correlation test are presented in Multimedia Appendix 3 [72]. Finally, the small sample size relative to the number of acoustic features, the use of imputing labels, and the Synthetic Minority Oversampling technique may contribute to overfitting. To mitigate the risk of overfitting, we carefully selected the features included in the classifier using filter methods and performed k-fold cross-validation.

### Future Perspective

Future studies that consider potential confounders may provide further insights into the factors that influence voice changes in patients with suicidal tendencies. Different fusion methods, such as ensemble modeling that incorporates voice text features or other behavioral markers that may be collected during clinical interviews, could also improve the classification algorithm. However, studies using voice as biomarkers must also consider the ethical implications of protecting the privacy and confidentiality of study participants [73].

The algorithms developed in this study have important implications for suicide prevention and telemedicine, as they can serve as the basis for the development of timely assessments and interventions for patients at a high risk of suicide. In particular, a longitudinal approach to voice analysis for identifying increased suicide risk can be used to monitor high-risk groups and intervene earlier. By using screening instruments based on acoustic features, which can be obtained remotely, such algorithms can identify patients who require urgent intervention such as immediate medication adjustment or hospitalization. Previous research has suggested that using a suicide risk detection tool based on speech can be useful for clinical decision-making, providing real-time risk assessments on a telehealth platform, and be a part of suicide preventive strategies in the future [74,75]. Future studies should focus on the implementation and validation of these tools in real-world clinical settings. It is worth noting that a study on telephone follow-up programs for suicide prevention concluded that longer programs lasting ≥12 months were more effective in preventing suicidality compared with shorter programs [76], highlighting the importance of longitudinal assessment and intervention for suicide prevention.

### Conclusions

To our knowledge, this is the first study to compare between-person and within-person classifications to detect high suicide risk based on acoustic voice characteristics. Our results indicate that a within-person model outperforms the between-person model, highlighting the importance of accounting for individual variability when analyzing acoustic features for predicting the clinical state. This study supports the use of longitudinal and personalized approaches to assess suicidality.

Ultimately, our work supports the possibility of developing a clinical decision support system based on an automated real-time quantitative analysis of voice features that could help screen high-risk patients for suicidality, which can be applied during telemedicine.

## Appendix

Multimedia Appendix 1 Confusion matrix and the receiver operating characteristic curve of classification between suicidal group versus nonsuicidal group, including healthy controls, by acoustic features.

Multimedia Appendix 2 The baseline characteristics of the high suicide risk and low suicide risk groups included healthy controls.

Multimedia Appendix 3 Correlation analysis between Beck Scale for Suicidal Ideation and other scales (Pearson *r* and 2-tailed *P* value).

## Abbreviations

ANN: artificial neural network
AUC: area under receiver operating characteristic curve
BAI: Beck Anxiety Inventory
BIS: Barratt Impulsiveness Scale
C-SSRS: Columbia Suicide Severity Rating Scale
HDRS: Hamilton Depression Rating Scale
HS: high suicide risk
light GBM: light gradient boost
MFCC: Mel-frequency cepstral coefficient
PHQ-9: Patient Health Questionnaire-9
RF: random forest
SES: socioeconomic status
SSI: Beck’s Scale for Suicidal Ideation
SVM: support vector machine
XGB: extreme gradient boot

## Data Availability

Data cannot be publicly provided because of privacy concerns. Written informed consent obtained from the study participants did not permit public sharing of the data. The principal investigator of the project (YMA) and the corresponding author (HL) had full access to the data and were responsible for its integrity. Further inquiries, including the study protocol, should be directed to these authors.
