# Supplementary figures and images for: Acoustic Analysis of Speech for Screening for Suicide Risk: Machine Learning Classifiers for Between- and Within-Person Evaluation of Suicidality
Source: J Med Internet Res. 2023 Mar 23;25:e45456. doi: 10.2196/45456 (PMC10131783; doi:10.2196/45456)

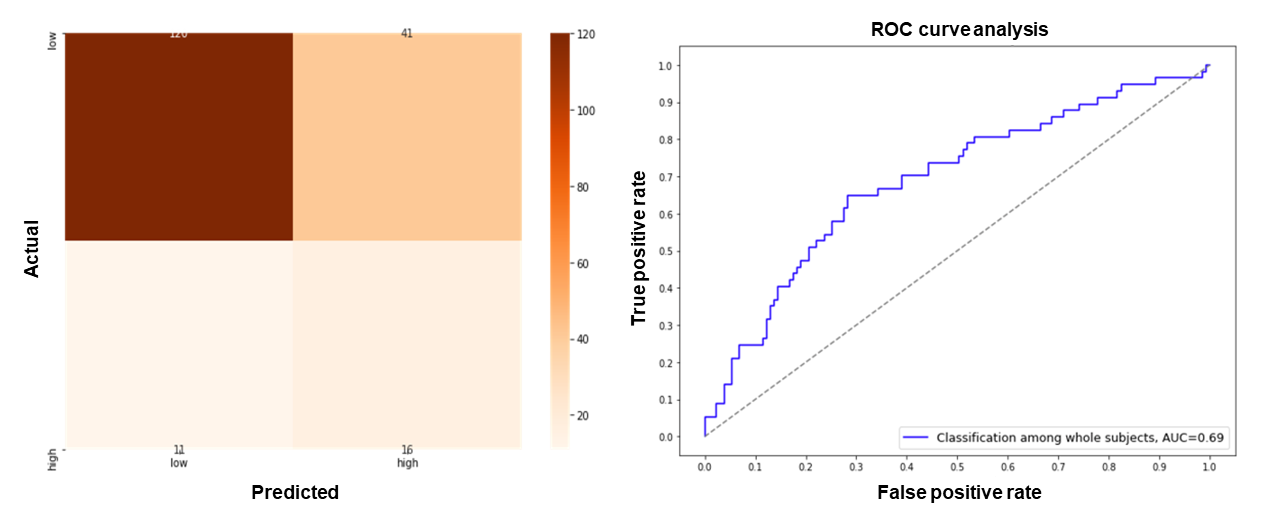

Supplement: Multimedia Appendix 1 [file jmir_v25i1e45456_app1.png]
