# Supplementary material for: Acoustic Analysis of Speech for Screening for Suicide Risk: Machine Learning Classifiers for Between- and Within-Person Evaluation of Suicidality
Source: J Med Internet Res. 2023 Mar 23;25:e45456. doi: 10.2196/45456 (PMC10131783; doi:10.2196/45456)
Supplement: Multimedia Appendix 2 [file jmir_v25i1e45456_app2.docx]

Supplementary table 2. Baseline characteristics of the HS group and LS group,
which includes healthy controls

|  | High risk of  suicide | Low risk of  suicide, including healthy controls | *P*-value |
| --- | --- | --- | --- |
| Number of subjects (%) | 57 (30) | 131 (70) |  |
| Age in years, mean (SD) | 29.61 (9.73) | 36.61 (11.54) | <.001 |
| Gender (M/F) | 13/44 | 24/107 | .609 |
| Body mass index, mean (SD) | 24.72 (4.87) | 22.95 (3.52) | .016 |
| Household income^a^, median (range) | 500(50-5000) | 500(74-1700) | .657 |
| Antipsychotics dosage^b^, median (range) | 7(0-85) | 0(0-70) | <.001 |
| PHQ-9^c^, mean (SD) | 17.42(5.80) | 3.40(4.99) | <.001 |
| HDRS^d^, mean (SD) | 17.93(4.49) | 6.85(5.42) | <.001 |
| BAI^e^, mean (SD) | 28.10(16.34) | 5.24(9.35) | <.001 |
| BIS^f^, mean (SD) | 63.81(7.85) | 63.88(8.03) | .106 |
| SSI^g^, mean (SD) | 23.37(5.78) | 2.71(3.82) | <.001 |

^a^Unit = 10,000 won; ^b^Antipsychotics dosage converted into the dose equivalent of aripiprazole; ^c^PHQ-9, Patient Health Questionnaire-9; ^d^HDRS, Hamilton Depression Rating Scale; ^e^BAI, Beck Anxiety Inventory; ^f^BIS, Barratt Impulsiveness Scale; ^g^SSI, Beck Scale for Suicidal Ideation.
